# TNF-α Directly Enhances Osteocyte RANKL Expression and Promotes Osteoclast Formation

**DOI:** 10.3389/fimmu.2019.02925

**Published:** 2019-12-13

**Authors:** Aseel Marahleh, Hideki Kitaura, Fumitoshi Ohori, Akiko Kishikawa, Saika Ogawa, Wei-Ren Shen, Jiawei Qi, Takahiro Noguchi, Yasuhiko Nara, Itaru Mizoguchi

**Affiliations:** Division of Orthodontics and Dentofacial Orthopedics, Department of Translational Medicine, Tohoku University Graduate School of Dentistry, Sendai, Japan

**Keywords:** osteocyte, TNF-α, RANKL, osteoimmunology, osteoclastogenesis

## Abstract

Osteoimmunology peeks into the interaction of bone and the immune system, which has largely proved to be a multiplex reaction. Osteocytes have been shown to regulate bone resorption through the expression of RANKL in physiologic and pathologic conditions. TNF-α, a product of the immune system, is an important cytokine regulating bone resorption in inflammatory conditions either directly or by increasing RANKL and M-CSF expressions by osteoblasts and stromal cells. The effect of TNF-α on a wide range of cell types has been documented; however, the direct effect of TNF-α on osteocytes has not been established yet. In this study, primary osteocytes were isolated by cell sorting from neonatal calvaria of Dmp1-Topaz mice, which express the green fluorescent protein under the influence of dentin matrix protein 1 promoter. The results show that osteocytes have a significantly higher RANKL mRNA expression when cultured with TNF-α. A co-culture system of osteocytes and TNF receptors I and II deficient osteoclast precursors treated with TNF-α show a significant increase in TRAP-positive cells while cultures without TNF-α failed to show TRAP-positive cells. Additionally, *in vivo* experiments of TNF-α injected to mouse calvaria show an increase in TRAP-positive cell number in the suture mesenchyme and an increase in the percentage of RANKL-positive osteocytes compared to PBS-injected calvaria. Osteocytes cultured with TNF-α show up-regulation of MAPKs phosphorylation measured by western blot, and adding MAPKs inhibitors to osteocytes cultured with TNF-α significantly decreases RANKL mRNA expression compared to osteocytes cultured with TNF-α alone. We also found that TNF-α activates the NF-κB pathway in osteocytes measured as a function of p65 subunit nuclear translocation. TNF-α directly affects osteocyte RANKL expression and increases osteoclastogenesis; our results demonstrate that osteocytes guard an important role in inflammatory bone resorption mediated by TNF-α.

## Introduction

The bone is a dynamic tissue, it is continuously broken down and built through the process of bone remodeling in which bone cell populations serve to achieve a balance between episodes of resorption and deposition (1). Osteoclasts, which descend from the hematopoietic stem cell lineage are the sole cells responsible for resorbing bone (2). In bone osteolytic diseases such as rheumatoid arthritis and periodontitis (3), the balance is lost and equilibrium shifts in favor of bone resorption.

Molecular signals act together with cellular components to regulate bone resorption, macrophage colony-stimulating factor (M-CSF) is the first in line to induce osteoclastogenesis by binding to its c-fms receptor to promote differentiation and maturation of osteoclast precursors (4). Receptor activator of nuclear factor κB Ligand (RANKL) is a member of the tumor necrosis factor superfamily and secreted by osteoblasts, bone marrow stromal cells (5), and lymphocytes (6). RANKL is obligatory for bone resorption (2). RANKL interacts with its receptor RANK on the surface of osteoclast precursors and drives their differentiation to bone-resorbing osteoclasts, and its effect is diminished by osteoprotegerin (OPG), a soluble decoy receptor secreted by osteoblasts and stromal cells (7). Tumor necrosis factor-α (TNF-α) is one of the most versatile cytokines, it is a product of the immune system secreted by macrophages, and its role in bone inflammatory diseases is well documented as pro-resorptive aiding in disease progression (8). TNF-α produces an array of cellular responses by binding to two receptors TNF receptor I (TNFR I) and TNF receptor II (TNFR II) (9). TNF-α stimulates osteoclastogenesis by increasing the production of M-CSF and RANKL in marrow stromal cells (10, 11) and osteoblasts (12) but other researchers reported a mechanism independent of RANKL in which TNF-α directly stimulates osteoclast formation in M-CSF-dependent bone marrow macrophage cultures treated with TNF-α (13), others reported that this happens when cells are primed with RANKL first (14).

Osteocytes are terminally differentiated osteoblasts which became embedded in their secreted matrix (15, 16) and account for 90% of the bone cell population, they reside within lacunae and communicate with each other and other cell types through processes extending to the bone surface (17). Osteocytes were shown to function in regulating mineral metabolism, remodeling the perilacunar matrix, and as a mechanosensory cell (18). Recently two groups demonstrated that osteocyte RANKL is the most important in physiologically supported osteoclastogenesis in the developing skeleton (19, 20). Osteocyte RANKL was shown to be important postnatally in osteocyte-specific RANKL deficient mice as these mice aged; an osteopetrotic phenotype became increasingly evident. Also, specific deletion of RANKL in osteocytes resulted in mice with a severe osteopetrotic phenotype and protection against loss of bone that accompanies unloading of mechanical force (20). Others reported that deletion of osteocyte RANKL confers protection against infection-induced periodontal bone loss (21) and an increase in cancellous bone mass in osteogenesis imperfecta mice (22). It is clear that osteocyte RANKL influences bone resorption both in health and disease.

An increasing body of research has linked TNF-α to osteocyte RANKL in inflammation-induced bone loss. A study where TNF-α antagonist was used resulted in a marked decrease in the number of RANKL-positive osteocytes and osteoclast formation in diabetic rats with periodontitis (23). Blockage of TNF-α in a co-culture of an osteocyte rich fraction and bone marrow cells treated with *Pasteurella multocida* toxin resulted in a decreased number of RANK positive cells, a marker of osteoclasts (24).

TNF-α and osteocyte RANKL are linked to inflammation-induced bone loss, but whether TNF-α has a direct effect on osteocytes is not clear. In this study, we provide evidence that TNF-α can directly affect osteocyte RANKL expression by activation of downstream MAPKs phosphorylation and induces osteocyte osteoclastogenic ability both *in vivo* and *in vitro*.

## Materials and Methods

### Mice and Reagents

Eight-week-old C57BL6/J wild-type (WT) were purchased from CLEA Japan Inc. (Tokyo, Japan). B6.129S-Tnfrsf1a^*tm*1*Imx*^ Tnfrsf1b^*tm*1*Imx*^/J (TNF receptor I, II deficient) mice and C57BL/6-Tg(Dmp1-Topaz)1Ikal/J mice were purchased from The Jackson Laboratory (Bar Harbor, ME, USA). All animal procedures were performed in accordance with Tohoku University regulations. Recombinant murine TNF-α was prepared in our laboratory as described previously (10). Recombinant mouse M-CSF was purified from an M-CSF expressing cell line (25).

### Isolation of Osteocytes

To isolate osteocytes of high purity, we followed the protocol published by Halleux et al. (26, 27) with few modifications. Neonatal calvariae of C57BL/6-Tg(Dmp1-Topaz)1Ikal/J mice which express the topaz variant of the green fluorescent protein (GFP) were enzymatically digested using 0.2% (w/v) collagenase (Wako, Osaka, Japan) and 5 mM ethylenediaminetetraacetic acid (EDTA) (Dojindo, Kumamoto, Japan) prepared with 0.1% BSA (Sigma-Aldrich, MO, USA) in PBS and filtered through a 0.2 μm filter. The collagenase was prepared fresh just before use in isolation buffer (70 mM NaCl, 10 mM NaHCO, 60 mM sorbitol, 3 mM K_2_HPO_4_, 1 mM CaCl_2_, 0.1% (w/v) BSA, 0.5% (w/v) glucose and 25 mM HEPES. Calvariae were incubated in collagenase for 20 min or EDTA for 15 min at 37°C on a shaker as follows: fraction 1 (collagenase), fraction 2 (EDTA), fraction 3 (collagenase), fraction 4 (collagenase), and fraction 5 (EDTA). Fractions 2 through 5 were collected and cultured in α-MEM (Wako, Osaka, Japan) containing 10% fetal bovine serum (FBS) (Biowest, Nuaillé, France), 100 IU/ml penicillin G, and 100 μg/ml streptomycin overnight. Fraction 2 was used in subsequent experiments as an osteoblast high fraction. Adherent cells were harvested using trypsin-EDTA (Life Technologies, NY, USA) and strained through a 40 μm nylon cell strainer (FALCON, NY, USA) in preparation for fluorescence-activated cell sorting in FACSAria^TM^ II (BD Biosciences, NJ, USA). GFP positive and negative cells were visualized using a fluorescence microscope (Olympus IX71, Tokyo, Japan) and the purity of osteocytes was determined through real-time RT-PCR.

### RNA Preparation and Real-Time RT-PCR Analysis

Osteocytes were cultured in α-MEM with or without TNF-α 100 ng/ml for 3 days. To test the effect of MAPKs inhibition, Osteocytes were pre-incubated with 10 μM of SB 203580 (InSolution™ SB 203580 – EMD Millipore, MA, USA) a p38 MAPK inhibitor, 10 μM of U0126 (InSolution™ U0126– EMD Millipore, MA, USA) a MEK1/2 inhibitor targeting ERK1/2 MAPK, and 10 μM of JNK Inhibitor II (InSolution™ JNK Inhibitor II- EMD Millipore, MA, USA) an inhibitor of JNK I, II, and III. Total RNA was obtained from lysed cells by using RNeasy Minikit (QIAGEN, Hilden, Germany) according to manufacturer's instructions. Total RNA was used to synthesize cDNA using the SuperScript^®^ IV First-Strand Synthesis System (Invitrogen™, CA, USA) according to the manufacturer's instructions. mRNA expression values were measured using the Thermal Cycler Dice Real-Time System (Takara, Shiga, Japan). Glyceraldehyde 3-phosphate dehydrogenase (GAPDH) was used as a reference gene. The following primers were used: RANKL (5′-CCCATCGGGTTCCCATAAAGTC-3′), (5′-GCCTGAAGCAAATGTTGGCGTA-3′), OPG (5′-ATCAGAGCCTCATCACCTT-3′), (5′-CTTAGGTCCAACTACAGAGGAAC- 3′), GAPDH (5'-GGTGGAGCCAAAAGGGTCA-3′), (5′-GGGGGCTAAGCAGTTGGT-3′), M-CSF(5′-TGATTGGGAATGGACACCTG-3′), (5′-AAAGGCAATCTGGCATGAAGT-3′). The purity of osteocytes obtained through cell sorting was checked against the following primers: Dmp1(5′-ACCACACGGACAGCAGTGAATC-3′), (5′-CCTCATCGCCAAAGGTATCATCTC-3′), SOST (5′-AGCCTTCAGGAATGATGCCAC-3′), (5′-CTTTGGCGTCATAGGGATGGT-3′), while osteoblast rich fraction content (fraction 2) was tested against Kera (5′-TCCCCCATCAACTTATTTTAGC-3′), (5′-GGTTGCCATTACAGGACCTT-3′).

### Preparation of Osteoclast Precursors and Co-culture System Set-Up

The bone marrow of long bones of WT and TNFR I, II deficient mice was flushed and cultured in α-MEM containing 10% FBS, 100 IU/ml penicillin G, 100 μg/ml streptomycin, and M-CSF 100 ng/ml for 4 days. Attached cells were harvested by trypsinization and used as osteoclast precursors. Osteocytes 2.5 × 10^4^/well were cultured overnight in α-MEM and harvested WT or TNFR I, II deficient osteoclast precursors 5 × 10^4^/well were added on top of osteocytes (2:1), and the co-culture was maintained either without M-CSF, with M-CSF alone (100 ng/ml), TNF-α alone (100 ng/ml), M-CSF+TNF-α (100 ng/ml), or M-CSF+RANKL 100 ng/ml in a 96 well plate. To test the effect of OPG on osteoclastogenesis, osteocytes and TNFR I, II deficient osteoclast precursors were co-cultured with M-CSF+TNF-α (100 ng/ml) or M-CSF+TNF-α+OPG (100 ng/ml), OPG was purchased from R&D Systems (MN, USA). To test the significance of cell-to-cell contact, an osteocyte-conditioned medium was prepared as follows: osteocytes were cultured with M-CSF+TNF-α (100 ng/ml) for 1 day, the medium was then aspirated and added to TNFR I, II deficient osteoclast precursor, as a positive control TNFR I, II deficient osteoclast precursor were cultured with M-CSF+RANKL (100 ng/ml). The co-cultures were maintained for 4 days after which they were terminated by fixation with 4% paraformaldehyde and stained with tartrate-resistant acid phosphatase (TRAP) staining consisting of acetate buffer (pH 5.0), naphthol AS-MX phosphate, fast red violet LB salt, and 50 mM sodium tartrate. Cells were considered osteoclasts if they were TRAP-positive and had 2 or more nuclei.

### Histological Examination

WT mice were subjected to subcutaneous supra-calvarial injection of either TNF-α 3.0 μg/100 μl or PBS for 5 consecutive days, on the 6th day the mice were sacrificed and the calvaria was excised. The calvaria was fixed in 4% paraformaldehyde overnight at 4°C and demineralized in 14% EDTA for 3 days at room temperature. The calvaria was trimmed from soft tissue and cut into 3 pieces perpendicular to the sagittal suture, and dehydrated then embedded in paraffin. Paraffin-embedded calvaria specimens were cut into 5 μm-thick sections using a microtome (Leica Biosystems, Wetzlar, Germany). Histological sections were deparaffinized, rehydrated, and blocked with 3% skimmed milk for 30 min at 37°C then stained with anti-RANKL antibody (FL-317 SCBT rabbit polyclonal IgG) diluted to 1:50 in can get immunostain solution B (Toyobo, Osaka, Japan) overnight at 4°C. Sections were washed and incubated with Histofine^®^ Simple Stain^TM^ Mouse MAX PO (R) (Nichirei Bioscience, Tokyo, Japan) for 1 h at room temperature then stained with hematoxylin as a counterstain. RANKL-positive osteocytes were counted as a percentage from the total number of osteocytes per 400 × 400 μm^2^ section area. For osteoclast formation examination, calvarial sections from WT mice were deparaffinized, rehydrated, and stained with TRAP staining then counter-stained with hematoxylin. TRAP-positive cells with two or more nuclei were counted and corrected to total bone surface area.

### Western Blot

Osteocytes were cultured in α-MEM containing 10% FBS, 100 IU/ml penicillin G, 100 μg/ml streptomycin overnight. Osteocytes were then cultured in a 24-well plate in α-MEM containing no serum (serum starvation) for 3 h. TNF-α 100 ng/ml was then added to the wells for specific periods (0, 5, 15, 30, 60) minutes. Control wells (0 min) had no TNF-α. Cells were lysed using radioimmunoprecipitation (RIPA) assay buffer (Millipore, MA, USA) containing 1% protease and phosphatase inhibitor (Thermo Fisher Scientific, IL, USA) on ice for 15 min; insoluble material was separated by centrifugation. Total protein was quantified using Pierce BCA protein assay kit (Thermo Fisher Scientific, IL, USA). Protein was treated with β-mercaptoethanol (BioRad, CA, USA) and laemmli sample buffer (BioRad, CA, USA) 1:1 and denatured at 95°C for 5 min as preparation for SDS-PAGE. Equal amounts of protein were loaded into gels 4–15% Mini-PROTEAN TGX Precast Gels (Bio-Rad, CA, USA) and transferred to a PVDF Trans-Blot Turbo Transfer System (Bio-Rad, CA, USA) then incubated in Block-Ace (DS Pharma Biomedical, Osaka, Japan) at 4°C overnight. Membranes were incubated with the following antibodies: rabbit monoclonal Phospho-p38 MAPK (Thr180/Tyr182), p38 MAPK rabbit Ab, rabbit monoclonal Phospho-p44/42 (ERK1/2) MAPK (Thr202/Tyr204), p44/42 (ERK1/2) MAPK rabbit Ab, polyclonal Phopho-SAP/JNK (Thr183/Tyr185), SAP/JNK MAPK rabbit Ab, polyclonal Phospho-AKT (Ser473), AKT rabbit Ab (Cell Signaling Technologies, MA, USA), mouse monoclonal anti-β-actin antibody (Sigma-Aldrich, MO, USA) at a dilution 1:1,000 overnight at 4°C. The membranes were washed in tris buffered saline with Triton X-100 (TBS-T) and incubated with horseradish peroxidase-conjugated anti-rabbit antibody (Cell Signaling Technologies, MA, USA) or anti-mouse antibody (GE Healthcare, IL, USA) at a dilution 1:5,000 for 1 h at room temperature. The signal was detected using an enhanced chemiluminescence detection system (SuperSignal West Femto Maximum Sensitivity Substrate, Thermo Fisher Scientific, IL, USA). Band density was measured using Image J (NIH).

### Preparation of Cells for Flow Cytometry

Bone marrow cells (BMC) from WT mice and fractions 2–5 from Dmp1-topaz mouse calvaria were suspended in α-MEM 100 IU/ml penicillin G, 100 μg/ml streptomycin. Cells were washed with 1% BSA in PBS, fixed in 4% paraformaldehyde for 3 min, and washed with 1% BSA in PBS 3 times. Cells were incubated with PE-conjugated anti-TNFR I antibody (CD120a mAb, GeneTex, GTX16198) at 1 μg/ml and PE-conjugated anti-TNFR II (CD120b mAb, BD Biosciences, 550086) at 1 μg/ml for 30 min on ice in the dark and washed 3 times with 1% BSA in PBS. Stained cells were analyzed with FACSAria^TM^ II (BD Biosciences, NJ, USA).

### Immunofluorescence

Osteoblasts from fraction 2 and osteocytes were cultured overnight in α-MEM 100 IU/ml penicillin G, 100 μg/ml streptomycin. Cells were washed with PBS, fixed with 4% formaldehyde for 15 min, washed 3 times with PBS and blocked with 3% BSA in PBS for 1 h. Cells were incubated with PE-conjugated anti-TNFR I antibody (CD120a mAb, GeneTex, GTX16198), and its isotype control (IgG2a isotype control mAb, GenTex, GTX35058) at 1 μg/ml and PE-conjugated anti-TNFR II (CD120b mAb, BD Biosciences, 550086), and its isotype control (IgG1λ1 isotype control mAb, BD Biosciences, 554711) at 1 μg/ml on ice in the dark for 1 h. Cells were washed 3 times with PBS before imaging. For testing the NF-κB pathway activation, osteocytes were cultured on collagen-coated (Nitta Gelatin Inc., Osaka, Japan) 8-chamber slide (Lab-Tek, Thermo Fisher Scientific, IL, USA) with or without TNF-α 100 ng/ml for 1 h. Cells were washed with PBS, fixed in 4% formaldehyde for 15 min and washed, then permeabilized with 0.1% Triton X-100 in PBS for 10 min, washed and blocked with 3% BSA in PBS for 1 h at room temperature (R/T). Cells were then incubated with anti-p65 antibody at (1:100) in 3% BSA (C-20 SCBT rabbit polyclonal IgG) at 4°C overnight. Cells were washed with PBS and incubated with Alexa Fluor 555 (goat anti-rabbit IgG) (1:400) in 3% BSA (Life Technologies, NY, USA) for 1 h in the dark at R/T, then washed and incubated with DAPI for 5 min. Fluorescent cell imaging was done using a fluorescence microscope (Olympus IX71, Tokyo, Japan).

### Statistical Analysis

The data were analyzed using student's *t*-test for two data sets and scheffe's test for multiple data sets. Data were presented as a mean ± standard deviation (SD). Results are representative of three or more independent experiments throughout the paper.

## Results

### Osteocyte Isolation and Characterization

**Figure 1:**
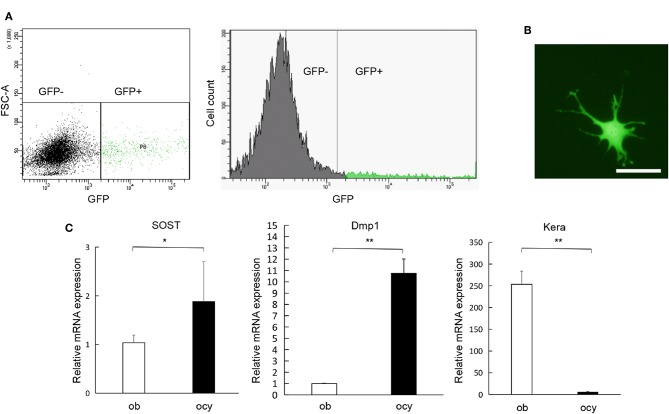
Isolation and characterization of osteocytes. **(A)** Separation of GFP-positive cells (osteocyte) and GFP-negative cells and the number of GFP-positive and GFP-negative cells obtained through fluorescent cell sorting. **(B)** Morphology of GFP-positive cells obtained from Dmp1-Topaz mice. **(C)** Expression levels of SOST, Dmp1, and Kera mRNA in GFP-positive cells and osteoblast rich fraction obtained through fractionation of neonatal Dmp1-Topaz calvaria (fraction 2) analyzed by qRT-PCR, ob = osteoblast, ocy = osteocyte. Scale bar = 50 μm, Data are expressed as mean ± SD. Statistical significance was determined by *t*-test (*n* = 4, ^*^P < 0.05, ^**^P < 0.01).

In order to extract osteocytes from their calcified bony matrix, we processed the calvaria using collagenase and EDTA, and then we run the digests through FACS to separate GFP-positive from GFP-negative populations (Figure 1A). The isolated cells had a characteristic osteocyte-like phenotype (Figure 1B). PCR analysis of the isolated cells using Dmp1, SOST and Kera confirmed that GFP-positive cells are highly expressive of Dmp1 and SOST which are genes highly expressed in osteocytes, GFP-negative cells showed the opposite with faint expression of Dmp1 and SOST and a significantly higher expression of Kera which is differentially expressed in osteoblasts (Figure 1C). This result confirms the successful separation of osteocytes from other cell populations.

### Osteocytes Express TNF Receptor I and TNF Receptor II

**Figure 2:**
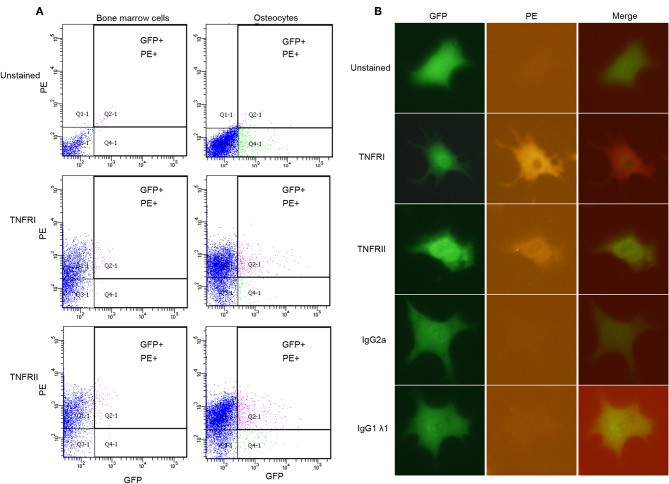
Osteocytes express TNFR I and TNFR II on their surface. **(A)** Flow cytometry analysis of osteocytes or bone marrow cells; unstained, stained with anti-TNFR I antibody, anti-TNFR II antibody. **(B)** Microscopic images of osteocytes obtained by immunofluorescence; unstained, stained with anti-TNFR I antibody, anti-TNFR II antibody, anti- IgG2a antibody (isotype control) and, anti-IgG1λ1 antibody (isotype control). *n* = 4. Images were processed using Image J (NIH) software.

Flow cytometry analysis shows that osteocytes (GFP+) stained for TNFR I and II express both receptors (Figure 2A). Unstained BMC population were used to account for background fluorescence, which shows that BMC population falls below the threshold level for both PE and GFP, while BMC stained for TNFR I and II used as positive controls confirm the expression of both receptors on their surface, and that osteocytes express both TNFR I and II on their surface. Results for immunofluorescence staining using osteocytes stained for TNFR I and II indicated that osteocytes express both receptors on their surface, unstained osteocytes and osteocytes stained with isotype controls do not show any fluorescent activity (Figure 2B). Osteoblasts stained for TNFR I and II also express both receptors while unstained osteoblasts and osteoblasts stained with isotype controls do not show fluorescent activity (Supplementary Figure 1).

### TNF-α Induces Osteocyte RANKL Expression *in vitro*

**Figure 3:**
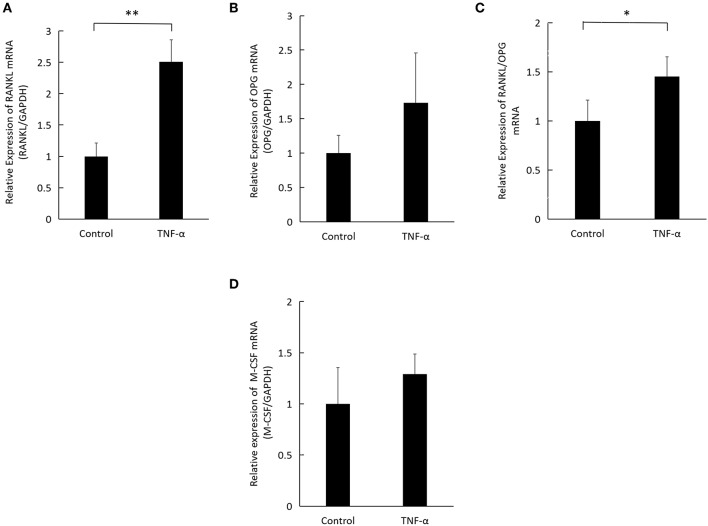
TNF-α induces RANKL expression in osteocytes. **(A)** Expression levels of RANKL mRNA, **(B)** OPG mRNA, **(C)** RANKL/OPG ratio, and **(D)** M-CSF mRNA in osteocytes, analyzed by real-time reverse transcription polymerase chain reaction (qRT-PCR). Total RNA was obtained from osteocytes cultured with TNF-α at 100 ng/ml for 3 days. Data are expressed as mean ± SD. Statistical significance was determined by *t*-test (*n* = 4, ^*^P < 0.05, ^**^P < 0.01).

We examined the change in RANKL, OPG, and M-CSF expression levels in osteocytes upon incubation of osteocytes with TNF-α for 3 days. PCR analysis revealed that osteocytes cultured with TNF-α expressed a significantly higher RANKL mRNA (Figure 3A) but no difference in OPG expression (Figure 3B). The ratio of RANKL/OPG was significantly higher in osteocytes cultured with TNF-α (Figure 3C), while M-CSF revealed no difference between osteocytes cultured with or without TNF-α (Figure 3D). The significantly higher ratio of RANKL/OPG indicates that TNF-α plays a role in osteocyte osteoclastogenic ability through supporting osteoclast precursor survival and differentiation.

### TNF-α Induced Osteocyte-Supported Osteoclast Formation in Co-culture

**Figure 4:**
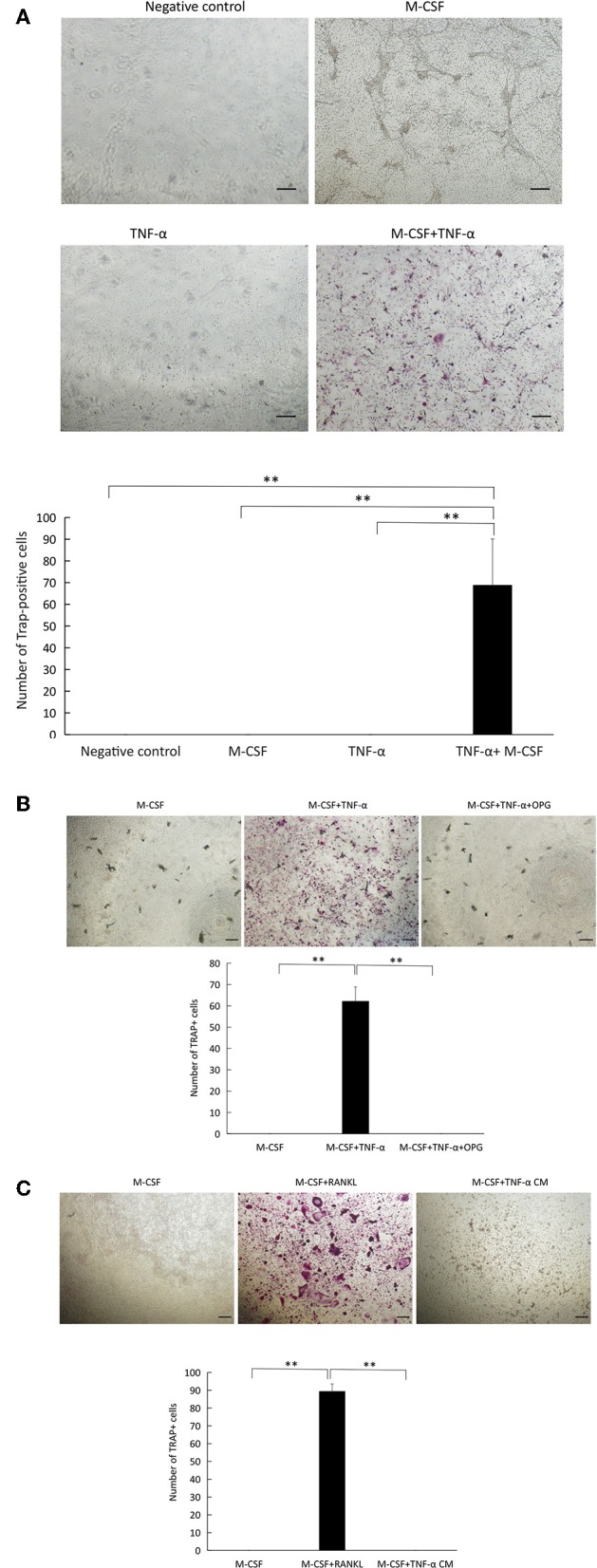
TNF-α supports osteocyte-induced osteoclastogenesis in co-culture. **(A)** Microscopic images and number of TRAP+ cells in co-cultures of osteocytes and TNFR I, II-deficient osteoclast precursors cultured alone, with or without M-CSF, TNF-α alone, with M-CSF +TNF-α (100 ng/ml). **(B)** Co-culture of osteocytes and TNFR I, II-deficient osteoclast precursors with M-CSF, and M-CSF+TNF-α with or without OPG (100 ng/ml). **(C)** TNFR I, II-deficient osteoclast precursors cultured in M-CSF, M-CSF+RANKL (100 ng/ml) or an osteocyte conditioned medium (CM) treated with M-CSF+TNF-α (100 ng/ml). Scale bar = 100 μm. Data are expressed as mean ± SD. Statistical significance was determined by scheffe's test (*n* = 4, ^**^P < 0.01).

To test whether osteocytes cultured with TNFR I, II-deficient osteoclast precursors in the presence of TNF-α successfully supports the generation of multinuclear TRAP-positive osteoclasts; we cultured osteocytes and TNFR I, II-deficient osteoclast precursor with TNF-α or without TNF-α in the presence of M-CSF. While osteocytes were able to induce osteoclast formation in TNF-α+M-CSF treated wells, osteoclastogenesis failed without the addition of M-CSF even with the addition of TNF-α (Figure 4A). Co-cultures of WT osteoclast precursor and osteocytes only supported osteoclast formation after adding M-CSF and either TNF-α or RANKL (Supplementary Figure 2). To test whether osteoclast formation in co-cultures treated with TNF-α is the result of RANKL expressed by osteocytes, we added OPG to co-cultures, which completely inhibited the formation of osteoclasts, this indicates that osteoclast formation was a result of RANKL expressed by osteocytes treated with TNF-α (Figure 4B). We also cultured TNFR I, II-deficient osteoclast precursor with an osteocyte conditioned medium treated with M-CSF+TNF-α, the conditioned medium failed to induce osteoclast formation, however adding RANKL to TNFR I, II osteoclast precursor induced osteoclast formation, which highlights the need for membrane-bound RANKL in osteocytes to induce osteoclastogenesis (Figure 4C).

### TNF-α Induces RANKL Expression in Osteocytes *in vivo*

**Figure 5:**
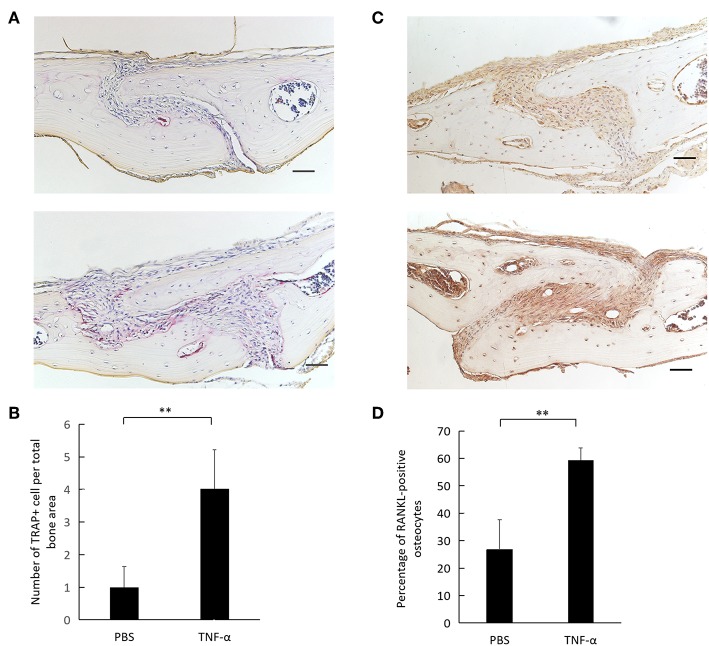
TNF-α induces osteoclastogenesis and osteocyte RANKL expression *in vivo*. **(A)** Histological sections of calvaria from WT mice after 5 consecutive days of TNF-α 3.0 μg/100 μl or PBS supracalvarial injection stained with TRAP staining and counterstained with hematoxylin. **(B)** Number of cells per total bone area. Cells were counted as osteoclasts if they were TRAP-positive and had multiple nuclei. **(C)** Histological sections of calvaria from WT mice after 5 consecutive days of TNF-α 3.0 μg/100 μl or PBS supracalvarial injection stained with anti-RANKL antibody and counterstained with hematoxylin, arrow = RANKL-positive osteocyte, arrow head = RANKL-negative osteocyte. **(D)** Percentage of RANKL-positive osteocytes over total osteocytes in a 400 × 400 μm^2^ bone area taken with the suture mesenchyme centered in the middle. Scale bar = 100 μm. Data are expressed as mean ± SD. Statistical significance was determined by *t*-test (*n* = 4, ^**^P < 0.01).

TRAP staining of calvaria revealed a higher number of osteoclasts in TNF-α injected calvaria compared to PBS injected calvaria (Figures 5A,B). Immunohistochemical examination of mouse calvaria injected with TNF-α revealed a higher expression of RANKL-positive osteocytes compared to calvaria injected with PBS (Figure 5C). The number of RANKL-positive osteocytes was significantly higher in the TNF-α injected group compared to the PBS injected group (Figure 5D).

### Activation of ERK1/2, P38 and JNK MAPKs by TNF-α Mediates RANKL Expression

**Figure 6:**
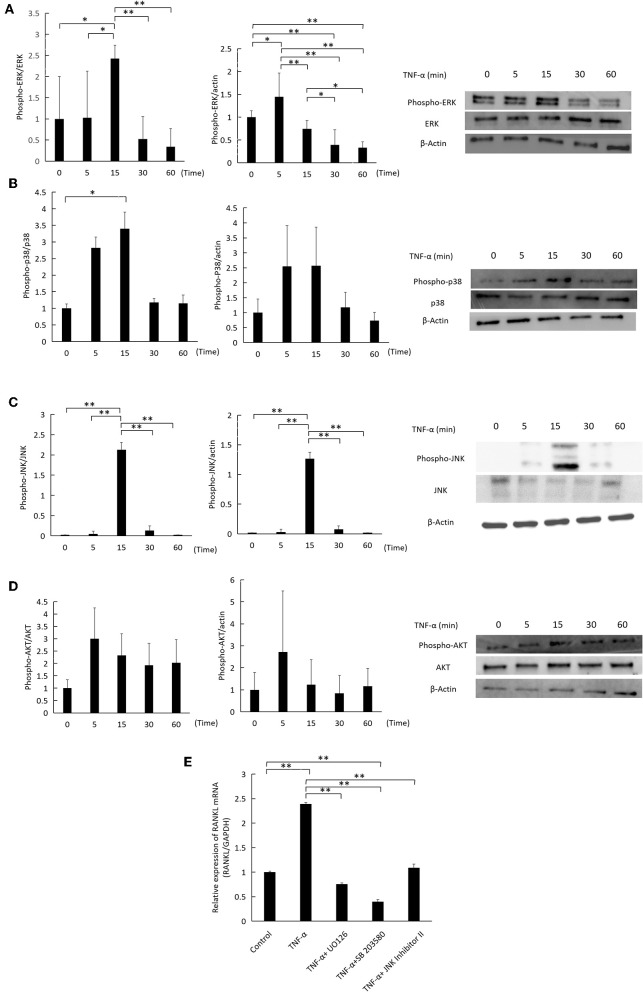
Effect of TNF-α on ERK1/2, P38, JNK MAPKs, and AKT phosphorylation in osteocytes. Osteocytes were incubated with TNF-α 100 ng/ml for 0, 5, 15, 30, 60 min. 0 indicates that no TNF-α was added. Cells were lysed and analyzed by western blotting, using antibodies for **(A)** phospho-ERK1/2, ERK1/2, **(B)** phospho-p38, p38 **(C)** phospho-JNK, JNK **(D)** phospho-AKT, AKT. β-actin was used as a loading control and band density was measured using Image J software, *n* = 3. **(E)** RANKL mRNA relative expression as measured by qRT-PCR. Osteocytes were cultured with TNF-α (100 ng/ml) and MAPKs inhibitors, U0126, SB 203580, and JNK inhibitor II (SP600125) at 10 μM for 3 days. Data are expressed as mean ± SD. Statistical significance was determined by scheffe's test (*n* = 3, ^*^P < 0.05, ^**^P < 0.01).

TNF-α was added to cultures of osteocytes for specific time intervals (0, 5, 15, 30, 60) minutes, while 0 indicates that no TNF-α was added. TNF-α transiently increased the phosphorylation of ERK1/2, p38, and JNK MAPKs in relation to the corresponding total protein and actin, peaking at 5 or 15 minutes (Figures 6A–C). There was no change in AKT phosphorylation (Figure 6D). Inhibition of ERK1/2, p38, and JNK MAPKs activation by U0126, SB 203580, and JNK inhibitor II, respectively, decreased RANKL mRNA expression significantly compared to cells treated with TNF-α only (Figure 6E).

### TNF-α Activates the NF-κB Signaling Pathway in Osteocytes

**Figure 7:**
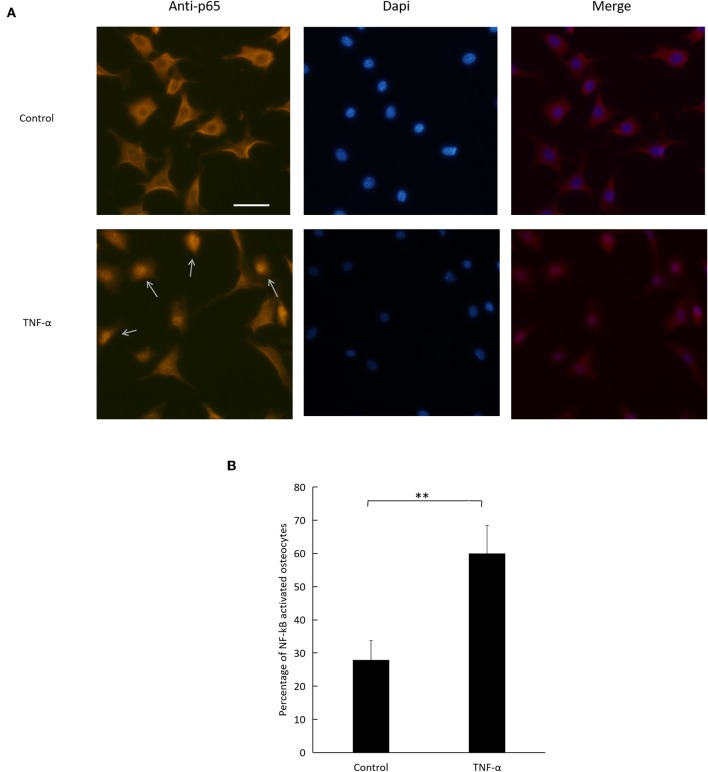
Effect of TNF-α on NF-κB pathway activation. **(A)** Fluorescent images of osteocytes cultured with or without TNF-α and stained for NF-κB p65 subunit. Arrows indicate NF-κB activated osteocytes. **(B)** Number of NF-κB activated osteocytes to total number of osteocytes imaged. Scale bar = 50 μm. Data are expressed as mean ± SD. Statistical significance was determined by *t*-test (*n* = 4, ^**^P < 0.01). Images were processed in Image J software.

To evaluate NF-κB pathway activation, we relied on immunofluorescence imaging of osteocytes cultured with TNF-α (Figure 7A). We assessed NF-κB p65 subunit nuclear localization and found that the number of activated osteocytes to total osteocyte number increased significantly in cells treated with TNF-α compared to control (Figure 7B).

## Discussion

Osteocytes have gained popularity ever since their isolation from bone became possible (28), from there osteocytes were shown to regulate mineral metabolism (29), remodel the extracellular matrix (30), act as an endocrine cell (31), a mechanosensory cell (32), and activate osteoclastogenesis through osteocyte apoptotic bodies (33). Recently, osteocyte-secreted RANKL has been considered as the most relevant to physiologic bone resorption (19, 34). However, it became clear that osteocyte RANKL is expressed in pathologic conditions as well, leading to bone damage in a range of diseases (34, 35), as do the findings of this study. In this study, we tested the direct effect of TNF-α, an important inflammatory cytokine on osteocytes and we found that TNF-α directly increases RANKL expression as well as osteocyte-aided osteoclastogenesis both *in vitro* and *in vivo*.

First, we obtained pure cultures of primary osteocytes by cell sorting. To test the purity of the isolated population, we selected Dmp1, a gene predominantly expressed by osteocytes and was reported to exhibit the highest expression level in osteocytes compared to osteoblasts, and Kera, a gene which encodes keratocan and has the highest change in negative expression as osteoblasts mature into their osteocytic phenotype, Dmp1 and Kera were reported to have the highest difference in their expression levels between osteocytes and osteoblasts amongst genes associated with extracellular matrix and secreted proteins (27).We also measured SOST expression which has been shown to be expressed in mature osteocytes as sclerostin, an antagonist for the Wnt/β-catenin pathway (18). Moreover, Dmp1 and SOST have been used as markers of osteocytes while Kera as a marker of osteoblasts (19), thus providing a rational for using the expression of Dmp1, SOST, and Kera to assess the success of osteocyte isolation.

Two cell surface receptors, p55 and p75 TNFRs mediate the action of TNF-α (36). We tested the expression of TNF receptors I and II on osteocyte surface using flow cytometry and immunofluorescent staining, the results of both of these experiments indicate the presence of both receptors on osteocyte surface, these data provided a basis for testing the direct effect of TNF-α on osteocytes bearing TNFR I and II when co-cultured with osteoclast precursors deficient in these two receptors. We set up a co-culture system of osteocytes and WT osteoclast precursors to check the effect of TNF-α or RANKL on osteoclast precursors and osteocytes when both are able to respond to TNF-α to confirm osteoclastogenesis. Then we used osteoclast precursor isolated from TNFR I, II deficient mice which do not respond to TNF-α due to the lack of TNFR I and II on their surface, while osteocytes were isolated from Dmp1-Topaz mice which respond to TNF-α, confirmed by the presence of TNFR I, II on their surface. This set-up allowed us to test the effect of TNF-α on osteocytes alone. Cells cultured with M-CSF and TNF-α showed an increase in TRAP-positive activity while cells cultured without TNF-α or with M-CSF alone did not show any TRAP-positive activity. Previous reports mentioned that TNF-α can directly induce osteoclastogenesis independent of RANKL (13), and to test whether TNF-α enhanced osteoclastogenesis due to an increase in RANKL expression or independent of it, we added OPG a decoy RANKL receptor which blocks RANKL/RANK interaction, and it completely inhibited the formation of osteoclasts, thus affirming that osteoclastogenesis mediated by TNF-α in osteocytes is the result of RANKL expression. One study reported that osteocyte have a 10x higher expression of RANKL mRNA compared to osteoblasts in an unstimulated state, however, in co-culture of osteocytes and osteoclast precursor, osteoclasts failed to appear unless stimulated with prostaglandin E_2_ (PGE_2_) and 1,25 dihydroxy vitamin D_3_ [1,25(OH)_2_D_3_], their conclusion was that despite the high expression of RANKL, downregulation of OPG expression is required for efficient osteoclastogenesis (19). Our result demonstrates that the increase in the ratio of RANKL/OPG in cultures stimulated with TNF-α is the result of a higher RANKL expression and not the downregulation of OPG expression. Furthermore, similar to published reports, a conditioned medium of osteocytes treated with TNF-α failed to support osteoclast formation, highlighting the importance of membrane-bound RANKL (19).

Osteocytes co-cultured with TNFR I, II deficient osteoclast precursor failed to form osteoclasts without M-CSF, even with the addition of TNF-α, which indicates that osteocytes are not a significant source of M-CSF. Our *in vitro* results also show that osteocytes cultured with TNF-α for 3 days exhibit no difference in M-CSF mRNA expression. Our interest in M-CSF expression in osteocytes stems from the findings of previous studies which noted that TNF-α increases the pool of osteoclast precursors available due to an increase in M-CSF production in stromal cells (11), and that M-CSF levels are increased in the synovial fluid around loose joint prosthesis (37) and in the serum of patients with rheumatoid arthritis (38), both of which exhibit a marked increase in TNF-α expression.

TNF-α was shown to increase the expression of RANKL in osteoblasts, stromal cells (5) and lymphocytes (6), and in this report we show that TNF-α increases osteocyte RANKL expression *in vivo* and *in vitro* by regulating MAPKs phosphorylation and possibly NF-κB activation. TNF-α is a potent activator of ERK, JNK and p38 MAPKs, and their interaction with TNF-α has functions upstream and downstream of TNF-α signaling (39). In this report, we tested the downstream inhibition of TNF-α action on ERK1/2, p38 and JNK MAPKs activation via selective U0126, SB203580 and a JNK inhibitor II (also known as SP600125) (40), respectively which attenuated RANKL mRNA expression in osteocytes treated with TNF-α.

NF-κB is involved in the transcriptional activation of inflammatory-related genes induced by TNF-α, and its activation is crucial for the protection of cells from apoptotic cell death induced by the TNFR I cytoplasmic death domain activation. In an unstimulated state, IκB proteins interact with NF-κB units to mask their nuclear translocation, in this report TNF-α significantly increased the nuclear presence of the NF-κB p65 unit in osteocytes nuclei after 1 h of treatment. AKT is also a pathway when activated works as an antiapoptotic signal, TNF-α stimulates the AKT pathway in a cell-type-specific manner (41), we found no difference in AKT phosphorylation, however whether the AKT pathway has a role in TNF-α signaling and RANKL expression in osteocytes or not needs further experimentation.

The results of this study can be summarized by asserting the role of osteocytes in their potential contribution to bone destruction in inflammatory bone diseases by increasing RANKL expression. Osteocytes could be a target therapeutic factor in future studies.

## Data Availability Statement

All datasets generated for this study are included in the article/Supplementary Material.

## Ethics Statement

The animal study was reviewed and approved by Animal Care and Use Committee of Tohoku University.

## Author Contributions

AM and HK contributed to conception, design, data acquisition, data analysis, data interpretation, and drafting of the manuscript. HK contributed to critical revision of the manuscript. FO, AK, SO, W-RS, JQ, TN, and YN collected the samples and performed data analyses. HK and IM supervised the project. All authors provided final approval and agreed to be accountable for all aspects of the work.

### Conflict of Interest

The authors declare that the research was conducted in the absence of any commercial or financial relationships that could be construed as a potential conflict of interest.

## Supplementary Material

Supplementary Figure 1 Microscopic images of osteoblasts obtained by immunofluorescence; unstained, stained with anti-TNFR I antibody, anti-TNFR II antibody, anti- IgG2a antibody (isotype control) and, anti-IgG1λ1 antibody (isotype control). *n* = 4. Images were processed using Image J (NIH) software.

Supplementary Figure 2 Microscopic images of co-cultures of osteocytes and WT osteoclast precursors treated with M-CSF, M-CSF+TNF-α, M-CSF+RANKL (100 ng/ml) and the number of TRAP+ multinuclear cells. Data are expressed as mean ± SD. Statistical significance was determined by scheffe's test (*n* = 4, ^**^*P* < 0.01).

## References

[1] Hadjidakis DJ Androulakis II. Bone remodeling. Ann N Y Acad Sci. (2006) 1092:385–96. 10.1196/annals.1365.035 17308163

[2] Teitelbaum SL. Bone resorption by osteoclasts. Science. (2000) 289:1504–8. 10.1126/science.289.5484.1504 10968780

[3] Crotti TN Dharmapatni AA Alias E Haynes DR. Osteoimmunology: major and costimulatory pathway expression associated with chronic inflammatory induced bone loss. J Immunol Res. (2015) 2015:281287. 10.1155/2015/281287 26064999 PMC4433696

[4] Hamilton JA. CSF-1 signal transduction. J Leukoc Biol. (1997) 62:145–55. 10.1002/jlb.62.2.145 9261328

[5] Suda T Takahashi N Udagawa N Jimi E Gillespie MT Martin TJ. Modulation of osteoclast differentiation and function by the new members of the tumor necrosis factor receptor and ligand families. Endocr Rev. (1999) 20:345–57. 10.1210/edrv.20.3.0367 10368775

[6] Kong YY Feige U Sarosi I Bolon B Tafuri A Morony S. Activated T cells regulate bone loss and joint destruction in adjuvant arthritis through osteoprotegerin ligand. Nature. (1999) 402:304–9. 10.1038/35005552 10580503

[7] Udagawa N Takahashi N Yasuda H Mizuno A Itoh K Ueno Y Osteoprotegerin produced by osteoblasts is an important regulator in osteoclast development and function^*^. Endocrinology. (2000) 141:3478–84. 10.1210/endo.141.9.7634 10965921

[8] Osta B Benedetti G Miossec P. Classical and paradoxical effects of TNF-alpha on bone homeostasis. Front Immunol. (2014) 5:48. 10.3389/fimmu.2014.00048 24592264 PMC3923157

[9] Sedger LM McDermott MF. TNF and TNF-receptors: From mediators of cell death and inflammation to therapeutic giants - past, present and future. Cytokine Growth Factor Rev. (2014) 25:453–72. 10.1016/j.cytogfr.2014.07.016 25169849

[10] Kitaura H Sands MS Aya K Zhou P Hirayama T Uthgenannt B. Marrow stromal cells and osteoclast precursors differentially contribute to TNF-alpha-induced osteoclastogenesis *in vivo*. J Immunol. (2004) 173:4838–46. 10.4049/jimmunol.173.8.4838 15470024

[11] Kitaura H. M-CSF mediates TNF-induced inflammatory osteolysis. J Clin Invest. (2005) 115:3418–27. 10.1172/jci26132 16294221 PMC1283943

[12] Hofbauer LC Lacey DL Dunstan CR Spelsberg TC Riggs BL Khosla S Interleukin-1β and tumor necrosis factor-α, but not interleukin-6, stimulate osteoprotegerin ligand gene expression in human osteoblastic cells. Bone. (1999) 25:255–9. 10.1016/s8756-3282(99)00162-3 10495128

[13] Kobayashi K Takahashi N Jimi E Udagawa N Takami M Kotake S. Tumor necrosis factor alpha stimulates osteoclast differentiation by a mechanism independent of the ODF/RANKL-RANK interaction. J Exp Med. (2000) 191:275–86. 10.1084/jem.191.2.275 10637272 PMC2195746

[14] Lam J Takeshita S Barker JE Kanagawa O Ross FP Teitelbaum SL. TNF-alpha induces osteoclastogenesis by direct stimulation of macrophages exposed to permissive levels of RANK ligand. J Clin Invest. (2000) 106:1481–8. 10.1172/JCI11176 11120755 PMC387259

[15] Franz-Odendaal TA Hall BK Witten PE. Buried alive: how osteoblasts become osteocytes. Dev Dyn. (2006) 235:176–90. 10.1002/dvdy.20603 16258960

[16] Holmbeck K Bianco P Pidoux I Inoue S Billinghurst RC Wu W. The metalloproteinase MT1-MMP is required for normal development and maintenance of osteocyte processes in bone. J Cell Sci. (2005) 118(Pt 1):147–56. 10.1242/jcs.01581 15601659

[17] Bonewald LF. Osteocytes as dynamic multifunctional cells. Ann NY Acad Sci. (2007) 1116:281–90. 10.1196/annals.1402.018 17646259

[18] Bonewald LF. The amazing osteocyte. J Bone Min Res. (2011) 26:229–38. 10.1002/jbmr.320 21254230 PMC3179345

[19] Nakashima T Hayashi M Fukunaga T Kurata K Oh-Hora M Feng JQ. Evidence for osteocyte regulation of bone homeostasis through RANKL expression. Nat Med. (2011) 17:1231–4. 10.1038/nm.2452 21909105

[20] Xiong J Piemontese M Onal M Campbell J Goellner JJ Dusevich V. Osteocytes, not osteoblasts or lining cells, are the main source of the RANKL required for osteoclast formation in remodeling bone. PLoS ONE. (2015) 10:e0138189. 10.1371/journal.pone.0138189 26393791 PMC4578942

[21] Graves DT Alshabab A Albiero ML Mattos M Corrêa JD Chen S. Osteocytes play an important role in experimental periodontitis in healthy and diabetic mice through expression of RANKL. J Clin Periodontol. (2018) 45:285–92. 10.1111/jcpe.12851 29220094 PMC5811370

[22] Zimmerman SM Heard-Lipsmeyer ME Dimori M Thostenson JD Mannen EM O'Brien CA. Loss of RANKL in osteocytes dramatically increases cancellous bone mass in the osteogenesis imperfecta mouse (oim). Bone Rep. (2018) 9:61–73. 10.1016/j.bonr.2018.06.008 30105276 PMC6077550

[23] Kim J-H Kim AR Choi YH Jang S Woo G-H Cha J-H. Tumor necrosis factor-α antagonist diminishes osteocytic RANKL and sclerostin expression in diabetes rats with periodontitis. PLOS ONE. (2017) 12:e0189702. 10.1371/journal.pone.0189702 29240821 PMC5730195

[24] Heni H Ebner J Schmidt G Aktories K Orth J. Involvement of osteocytes in the action of pasteurella multocida toxin. Toxins. (2018) 10:328. 10.3390/toxins10080328 30104531 PMC6115833

[25] Takeshita S Kaji K Kudo A. Identification and characterization of the new osteoclast progenitor with macrophage phenotypes being able to differentiate into mature osteoclasts. J Bone Min Res. (2000) 15:1477–88. 10.1359/jbmr.2000.15.8.1477 10934646

[26] Halleux C Kramer I Allard C Kneissel M. Isolation of mouse osteocytes using cell fractionation for gene expression analysis. In: Helfrich MH Ralston SH, editors. Bone Research Protocols. Totowa, NJ: Humana Press (2012). p. 55–66. 10.1007/978-1-61779-415-5_5 22130922

[27] Paic F Igwe JC Nori R Kronenberg MS Franceschetti T Harrington P. Identification of differentially expressed genes between osteoblasts and osteocytes. Bone. (2009) 45:682–92. 10.1016/j.bone.2009.06.010 19539797 PMC2731004

[28] van der Plas A Nijweide PJ. Isolation and purification of osteocytes. J Bone Min Res. (1992) 7:389–96. 10.1002/jbmr.5650070406 1609628

[29] Feng JQ Ward LM Liu S Lu Y Xie Y Yuan B. Loss of DMP1 causes rickets and osteomalacia and identifies a role for osteocytes in mineral metabolism. Nat Genet. (2006) 38:1310–5. 10.1038/ng1905 17033621 PMC1839871

[30] Lane NE Yao W Balooch M Nalla RK Balooch G Habelitz S Glucocorticoid-treated mice have localized changes in trabecular bone material properties and osteocyte lacunar size that are not observed in placebo-treated or estrogen-deficient mice. J Bone Min Res. (2005) 21:466–76. 10.1359/jbmr.051103 PMC1797152 16491295

[31] Dallas SL Prideaux M Bonewald LF The osteocyte: an endocrine cell … and more. Endoc Rev. (2013) 34:658–90. 10.1210/er.2012-1026 PMC3785641 23612223

[32] Bonewald LF. Mechanosensation and transduction in osteocytes. Bonekey Osteovision. (2006) 3:7–15. 10.1138/20060233 17415409 PMC1847717

[33] Kogianni G Mann V Noble BS. Apoptotic bodies convey activity capable of initiating osteoclastogenesis and localized bone destruction. J Bone Min Res. (2008) 23:915–27. 10.1359/jbmr.080207 18435576

[34] Metzger CE Narayanan A Zawieja DC Bloomfield SA. Inflammatory bowel disease in a rodent model alters osteocyte protein levels controlling bone turnover. J Bone Miner Res. (2017). 10.1002/jbmr.3027 27796050

[35] Kim J-H Lee D-E Cha J-H Bak E-J Yoo Y-J. Receptor activator of nuclear factor-κB ligand and sclerostin expression in osteocytes of alveolar bone in rats with ligature-induced periodontitis. J Periodontol. (2014) 85:e370–e8. 10.1902/jop.2014.140230 25070541

[36] Heller RA Krönke M. Tumor necrosis factor receptor-mediated signaling pathways. J Cell Biol. (1994) 126:5–9. 10.1083/jcb.126.1.5 8027185 PMC2120084

[37] Takei I Takagi M Ida H Ogino T Santavirta S Konttinen YT. High macrophage-colony stimulating factor levels in synovial fluid of loose artificial hip joints. J Rheumatol. (2000) 27:894–9. 10782812

[38] Yang P-T Kasai H Xiao W-G Zhao L-J He L-M Yamashita A. Increased expression of macrophage colony-stimulating factor in ankylosing spondylitis and rheumatoid arthritis. Ann Rheum Dis. (2006) 65:1671–2. 10.1136/ard.2006.054874 17105859 PMC1798478

[39] Sabio G Davis RJ. TNF and MAP kinase signalling pathways. Semin Immunol. (2014) 26:237–45. 10.1016/j.smim.2014.02.009 24647229 PMC4099309

[40] English JM Cobb MH. Pharmacological inhibitors of MAPK pathways. Trends Pharmacol Sci. (2002) 23:40–5. 10.1016/s0165-6147(00)01865-4 11804650

[41] Wajant H Pfizenmaier K Scheurich P. Tumor necrosis factor signaling. Cell Death Differ. (2003) 10:45–65. 10.1038/sj.cdd.4401189 12655295

