# Supplementary figures and images for: TNF-α Directly Enhances Osteocyte RANKL Expression and Promotes Osteoclast Formation
Source: Front Immunol. 2019 Dec 13;10:2925. doi: 10.3389/fimmu.2019.02925 (PMC6923682; doi:10.3389/fimmu.2019.02925)

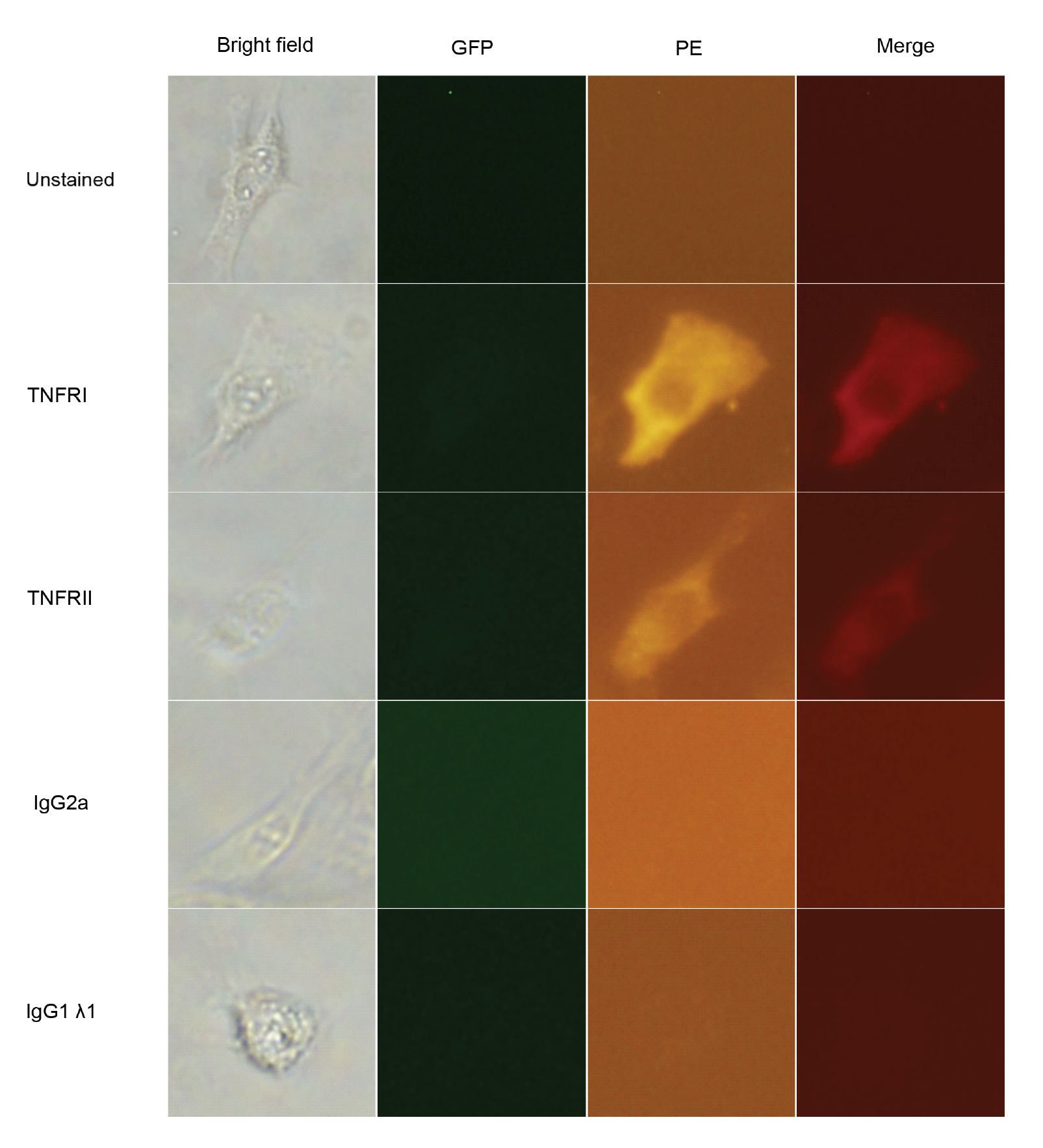

Supplement: Supplementary Figure 1 — Microscopic images of osteoblasts obtained by immunofluorescence; unstained, stained with anti-TNFR I antibody, anti-TNFR II antibody, anti- IgG2a antibody (isotype control) and, anti-IgG1λ1 antibody (isotype control). n = 4. Images were processed using Image J (NIH) software. [file Image_1.TIF]

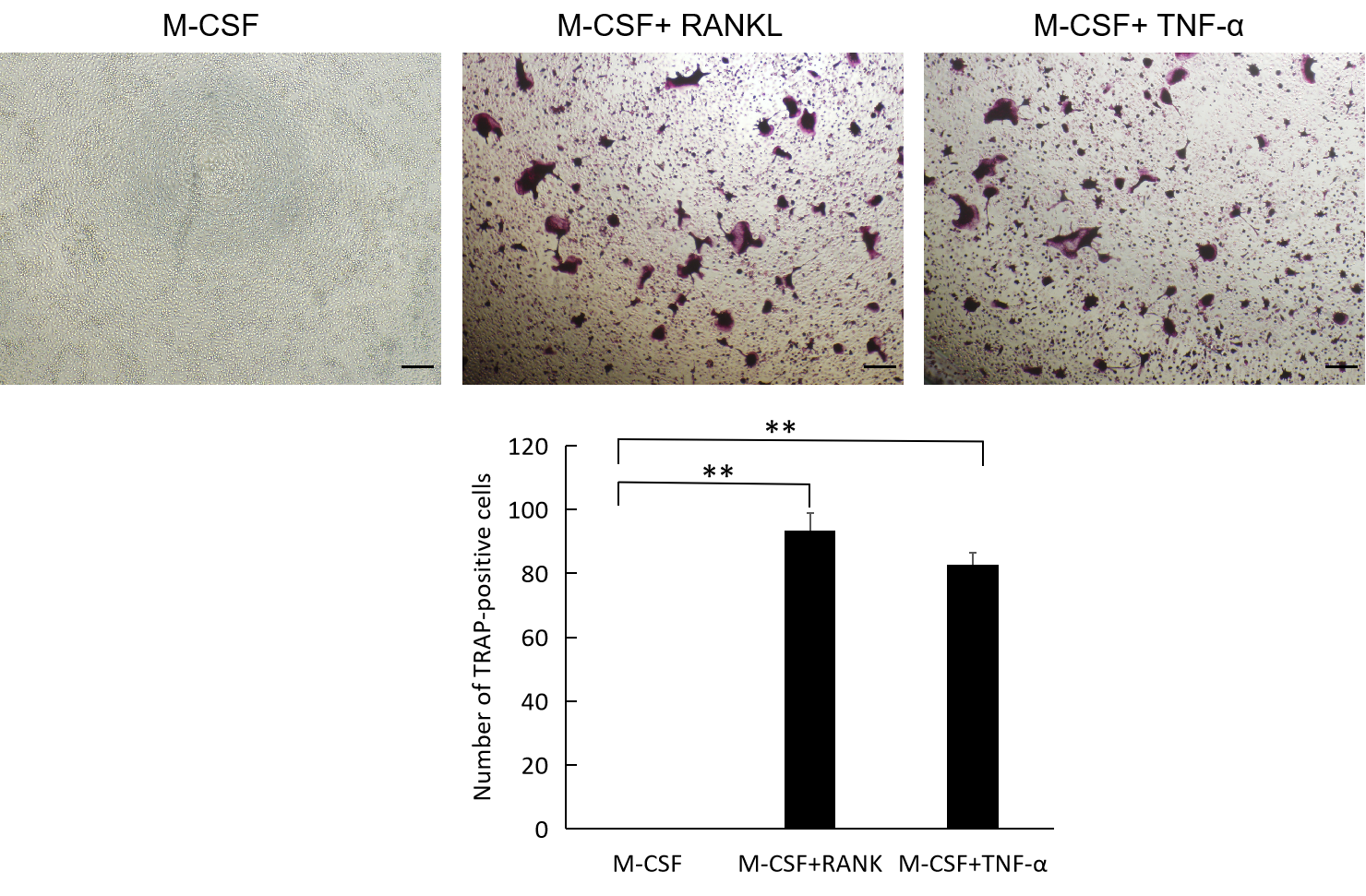

Supplement: Supplementary Figure 2 — Microscopic images of co-cultures of osteocytes and WT osteoclast precursors treated with M-CSF, M-CSF+TNF-α, M-CSF+RANKL (100 ng/ml) and the number of TRAP+ multinuclear cells. Data are expressed as mean ± SD. Statistical significance was determined by scheffe's test (n = 4, **P < 0.01). [file Image_2.TIF]
